# Mitofilin and CHCHD6 physically interact with Sam50 to sustain cristae structure

**DOI:** 10.1038/srep16064

**Published:** 2015-11-04

**Authors:** Chengli Ding, Zhifei Wu, Lei Huang, Yajie Wang, Jie Xue, Si Chen, Zixin Deng, Lianrong Wang, Zhiyin Song, Shi Chen

**Affiliations:** 1 Key Laboratory of Combinatorial Biosynthesis and Drug Discovery, Ministry of Education, School of Pharmaceutical Sciences, and Medical Research Institute, Wuhan University, Wuhan, China; 2 Taihe Hospital, Hubei University of Medicine, Shiyan, Hubei, China; 3 College of Life Sciences, Wuhan University, Wuhan, China

## Abstract

The inner mitochondrial membrane (IMM) invaginates to form cristae and the maintenance of cristae depends on the mitochondrial contact site (MICOS) complex. Mitofilin and CHCHD6, which physically interact, are two components of the MICOS. In this study, we performed immunoprecipitation experiments with Mitofilin and CHCHD6 antibodies and identified a complex containing Mitofilin, Sam50, and CHCHD 3 and 6. Using transcription activator-like effector nucleases (TALENs), we generated knockdown/knockout clones of Mitofilin and CHCHD6. Transmission electron microscopy (TEM) revealed that vesicle-like cristae morphology appeared in cell lines lacking Mitofilin, and mitochondria exhibited lower cristae density in CHCHD6-knockout cells. Immunoblot analysis showed that knockdown of Mitofilin, but not knockout of CHCHD6, affected their binding partners that control cristae morphology. We also demonstrated that Mitofilin and CHCHD6 directly interacted with Sam50. Additionally, we observed that Mitofilin-knockdown cells showed decreased mitochondrial membrane potential (ΔΨm) and intracellular ATP content, which were minimally affected in CHCHD6-knockout cells. Taken together, we conclude that the integrity of MICOS and its efficient interaction with Sam50 are indispensable for cristae organization, which is relevant to mitochondrial function.

Mitochondria are dynamic organelles with various functions. In addition to their role in energy generation, they are also closely involved in the calcium homeostasis, stress response and cell death pathways. Mitochondria consist of two membranes: the outer mitochondrial membrane (OMM) and the inner mitochondrial membrane (IMM). The IMM is a heterogeneous structure composed of morphologically distinct subdomains, including the inner boundary membrane (IBM), which faces the OMM, and the cristae membrane (CM), which protrudes into the matrix space. The connections between the IBM and the CM have been termed cristae junctions (CJs)^1^, and cytochrome *c* is separated from the intermembrane space (IMS) by the narrow CJs. The mitochondrial CM is the site of oxidative phosphorylation and harbors supercomplexes of the electron transport chain (ETC) and the F_1_F_0_-ATP synthase^2^,^3^. Morphological changes in CM domains have been observed in numerous pathologies^4^,^5^,^6^.

The OMM and IBM are connected by a multi-subunits complex called the mitochondrial contact site and cristae organizing system (MICOS)^7^. The MICOS complex consists of Mitofilin, Mio10, Mio27, Aim5, Aim13 and Aim37 in fungi. In human mitochondria, the MICOS complex is described to include MINOS1, Mitofilin (MINOS2), CHCHD3 (MINOS3) and CHCHD6 (CHCM1)^8^. Mitochondria in MICOS-deficient cells show disrupted cristae structures; nearly no CJs were observed in yeast cells lacking Fcj1 and Mio10^9^, and knockdown of mammalian MICOS components has been reported to result in altered cristae morphology^10^,^11^,^12^.

In addition to its role in inner membrane architecture, MICOS forms contact sites with the OMM to promote mitochondrial protein import into the OMM and IMS^7^. Most preproteins enter mitochondria through the translocase of the TOM complex in the OMM. They are then transported by the TIM22 and TIM23 complex to the mitochondrial matrix or the IMM or by the mitochondrial intermembrane space assembly machinery (MIA) pathway to the IMS. The sorting and assembly machinery (SAM)/translocase of outer membrane β-barrel proteins (TOB) complex (SAM/TOB complex) in the OMM is responsible for assembling β-barrel proteins into the OMM^13^. The SAM/TOB complex in mammalian mitochondria is composed of Sam50 and two other subunits, Metaxin 1 and Metaxin 2^14^,^15^,^16^. The interaction of Mitofilin with the TOM complex promotes protein import into the IMS via the MIA pathway^9^. Several reports found that Mitofilin physically interacts with the SAM/TOB complex of the OMM, which is required for the biogenesis of outer membrane β-barrel proteins^17^,^18^. Mitofilin, a core component of MICOS, has been described to interact with several other proteins such as Coiled-coil helix coiled-coil helix domain-containing protein 3 and 6 (CHCHD3 and CHCHD6), Sam50, Metaxin 1 and 2 and DnaJC11^19^, suggesting its involvement in mitochondrial protein import.

It remains unclear how the components of MICOS play roles in cristae organization. Sam50 was found to interact with Mitofilin and CHCHD3 to form the mitochondrial intermembrane space bridging (MIB) complex, which is crucial for the maintenance of cristae and assembly of respiratory chain complexes^20^. Sam50 depletion causes complete loss of cristae without affecting Mitofilin, and CHCHD 3 and 6^20^, suggesting that Sam50 is an important contact site for MICOS in the OMM.

In this study, we investigated the functions of Mitofilin and CHCHD6 in the preservation of mitochondrial cristae structure. We showed that stably knocking down Mitofilin leads to vesicle-like cristae structures and that knocking out CHCHD6 results in abnormal cristae with reduced cristae content. Mitofilin knockdown destabilizes MICOS, with drastic reductions in its components, whereas CHCHD6 knockout does not affect the levels of other MICOS protein components. Our results further revealed that both Mitofilin and CHCHD6 physically interact with Sam50. In addition, we found that knockdown of Mitofilin but not knockout of CHCHD6, resulted in apparent mitochondrial function abnormality. These results indicate that the integrity of MICOS and its efficient interaction with Sam50 are indispensable for cristae organization, which is relevant to mitochondrial function.

## Results

### Mitofilin, Sam50, and CHCHD 3 and 6 are in the same complex involved in regulating cristae structure

**Figure 1:**
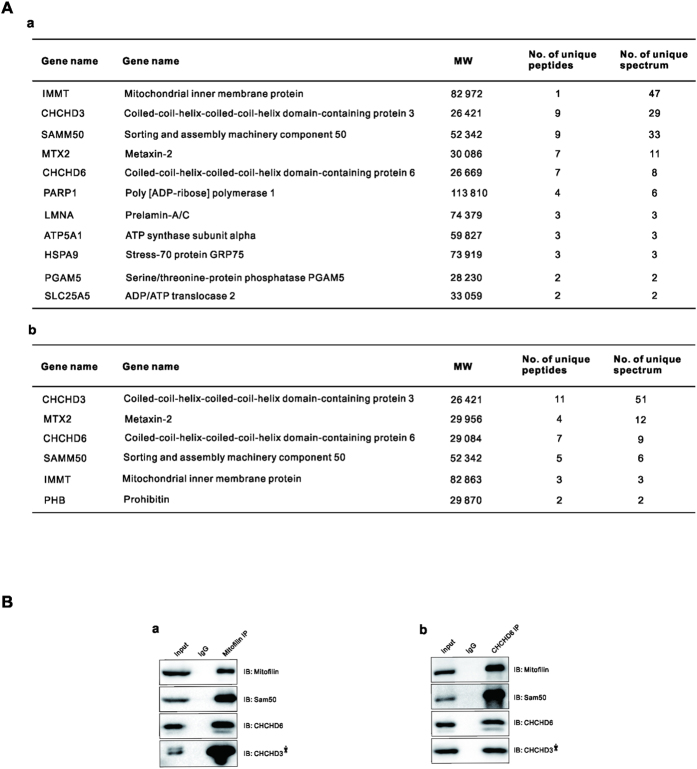
Mitofilin, Sam50, and CHCHD 3 and 6 are in the same complex, (**A**) a and b show MS data for the indicated proteins from the Mitofilin and CHCHD6 IPs. The number of total peptides (Total) and total unique peptides (Unique) identified by MS are shown. (**B**) The IP sample of endogenous Mitofilin (**a**) and CHCHD6 (**b**) were analyzed via SDS-PAGE followed by immunoblotting (IB) with indicated antibodies. *Asterisks* indicated that the blot of CHCHD3 was cropped from a different gel, as CHCHD3 and CHCHD6 have similar sizes. Full-length blots/gels are presented in Supplementary Figure 3.

Mitofilin is an abundant, conserved coiled-coil protein that is anchored to the IMM, and the bulk of its coiled-coil structure is exposed to the IMS^10^. CHCHD6 is a coiled-coil helix-coiled-coil helix (CHCH) IMM protein that physically interacts with Mitofilin^12^. To investigate the roles of the Mitofilin/MICOS complex, we focused on Mitofilin and CHCHD6. To investigate the MICOS interactome, we used Mitofilin and CHCHD6 antibodies to perform immunoprecipitation (IP) experiments in HeLa cells and analyzed the interactome by MS/MS sequencing. MS/MS data (Fig. 1A - a and – b; Supplementary Table S1) showed that the overlapped members from the two IP samples include Mitofilin, Sam50, and CHCHD 3 and 6, which indicates that these proteins are in the same complex. The IP results were then confirmed by immunoblotting with the corresponding antibodies (Fig 1. B - a and - b).

### Targeted gene knockdown of Mitofilin and knockout of CHCHD6

**Figure 2:**
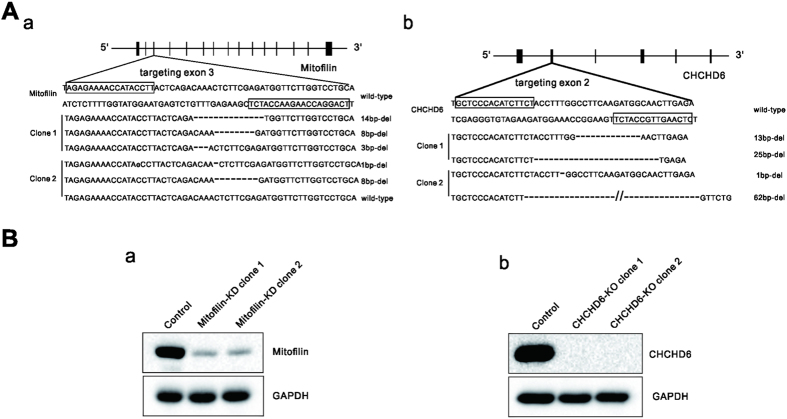
Generation of Mitofilin-knockdown and CHCHD6-knockout HeLa cell clones with TALENs (**A**) The boxes indicate the TALEN binding sites for Mitofilin, targeting exon 3 (**a**), and for CHCHD6, targeting exon 2 (**b**).Deletions in alleles of each clone are indicated. (**B**) Immunoblot analysis using whole-cell lysates from wild-type (WT) cells and the two Mitofilin-knockdown (a) or two CHCHD6-knockout (**b**) HeLa cell lines. Full-length blots/gels are presented in Supplementary Figure 4.

Transcription activator-like effector nucleases (TALENs) are a new class of engineered nucleases for genome editing that, due to their modular domain structure, are easier to design and construct than other types of nucleases^21^. TALEN binding pairs for genome editing are designed to target specific genes and create double-strand breaks, which are then repaired by nonhomologous end-joining to yield out-of-frame deletions or insertions^22^,^23^. To investigate the detailed roles of MICOS, we sought to knockout Mitofilin and CHCHD6, which are core and novel components of MICOS, respectively, in HeLa cells. We targeted the Mitofilin and CHCHD6 genes and generated one TALEN binding pair for each (Fig. 2A – a and – b). HeLa cells were transfected with plasmids encoding the TALEN binding pairs. Cells were then harvested to extract genomic DNA 48 h after transfection, and sequencing of the genomic DNA for Mitofilin or CHCHD6 showed that the designed TALEN binding pairs were efficient (Supplementary Fig. S1). Clonal cell populations were isolated and then screened by immunoblot analysis using the corresponding antibodies (see “Materials and Methods” for additional information). After screening 253 clones, we did not obtain any clones with homozygous frameshift Mitofilin mutations. However, two Mitofilin knockdown clones were obtained and confirmed by immunoblotting (Fig. 2B – a,b). For CHCHD6, we obtained two knockout cell clones with homozygous frameshift mutations. Sequencing and immunoblot results for the two CHCHD6 knockout clones are shown (Fig. 2A – b and B – b).

### Stable depletion of Mitofilin and knockout of CHCHD6 result in altered cristae structures

**Figure 3:**
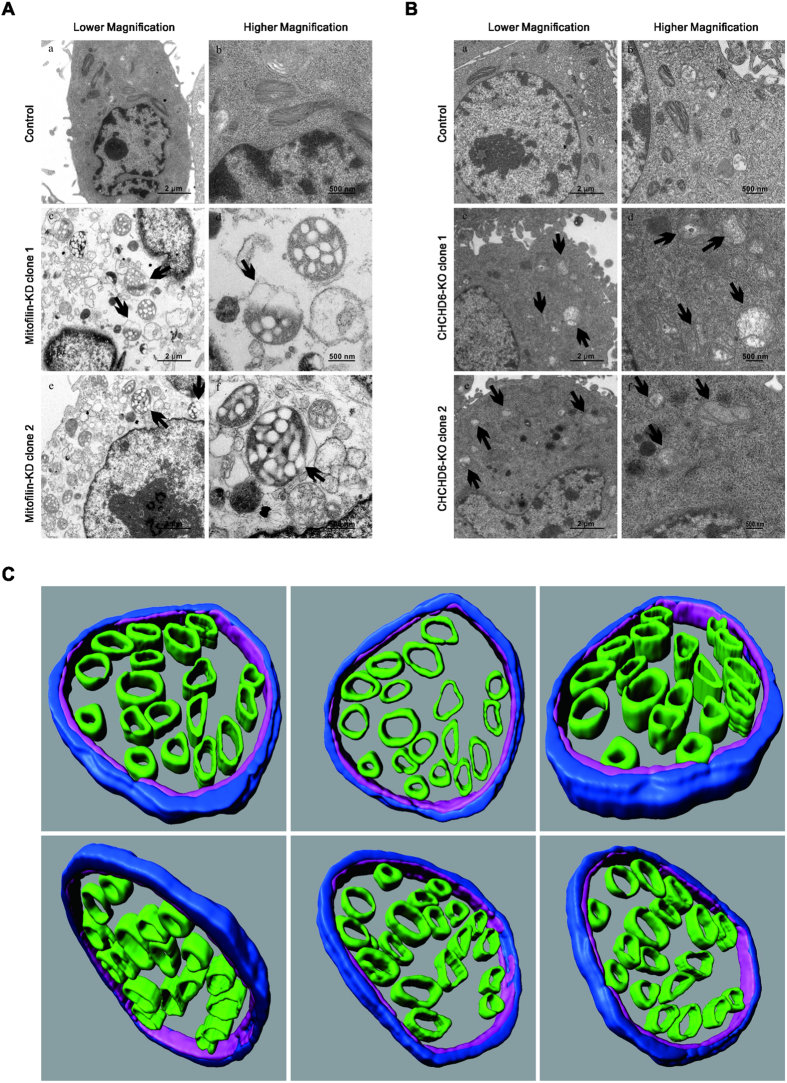
Altered cristae morphology caused by Mitofilin knockdown or CHCHD6 knockout. (**A**,**B**) Electron microscopy of mitochondria in control and Mitofilin-knockdown (**A**) or CHCHD6-knockout cells (**B**). The black arrows indicate disrupted mitochondria. (**C**) Electron tomography of mitochondrial morphological changes in Mitofilin-knockdown cells. The OMM is depicted in light blue, the IBM is shown in pink, and cristae are shown in green. These images are rotations of surface-rendered views of tomographic reconstructions of mitochondria.

To investigate the cristae structures in cells with stable depletion of Mitofilin and knockout of CHCHD6, two independent Mitofilin-deficient clones, two CHCHD6 knockout cell clones and control cells were subjected to TEM. Mitochondria in the Mitofilin-knockdown cells showed disruptions distinct from those observed in cells with transient Mitofilin knockdown via RNAi. Mitochondria in stable Mitofilin-knockdown cells displayed vesicle-like cristae (Fig. 3A), which is completely different from the onion-like cristae observed in Mitofilin-RNAi cells^10^,^24^. Compared with the normal narrow pleomorphic cristae (Fig. 3A - a, b), few cristae junctions could be observed (Fig. 3A - c, d, e, f). Some mitochondria in Mitofilin-knockdown cells exhibited a swollen morphology and even ruptured OMMs (Fig. 3A - c, d, e, f). To further analyze the altered morphology in Mitofilin-deficient cells, we assessed the IMM structure using tomographic three-dimensional reconstructions. The majority of the mitochondria in Mitofilin-deficient cells contained numerous cristae vesicles, and the matrix spaces appeared to be divided into compartments that were interconnected by the IMM (Fig. 3C). In CHCHD6-knockout HeLa cells, mitochondria appeared to have fewer CJs and a lower cristae density than control cells (Fig. 3B), which are different from the phenotypes described in other cell lines with CHCHD6 knocked down^12^.

### Mitofilin knockdown, but not CHCHD6 knockout, reduces the levels of proteins that regulate cristae morphology

**Figure 4:**
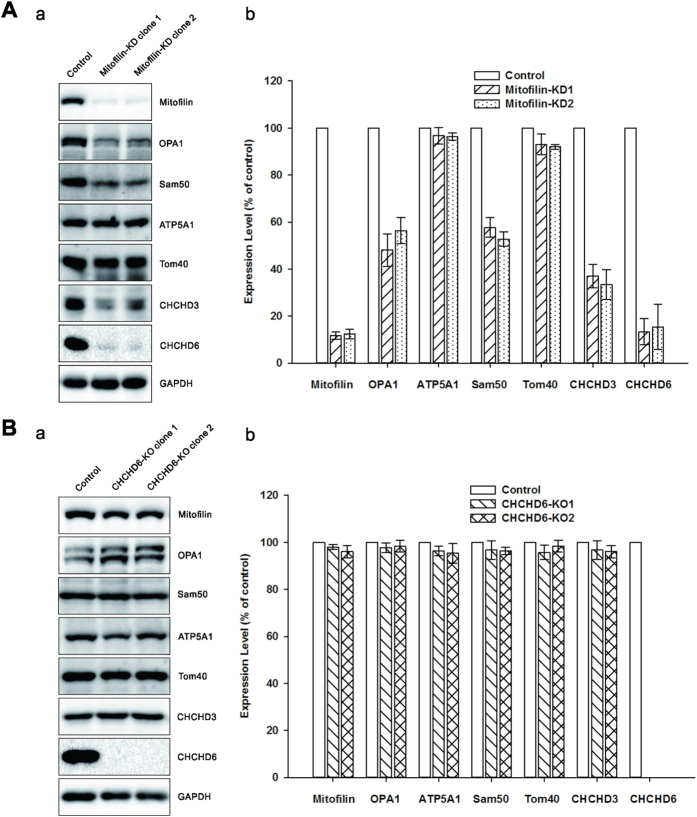
Steady-state levels several MICOS components in Mitofilin-knockdown or CHCHD6-knockout cells. Equal amounts of protein samples in control cells and Mitofilin knockdown cells (**A**) or CHCHD6 knockout cells (**B**) were analyzed via SDS-PAGE followed by immunoblot with indicated antibodies. The values represent the average protein expression ± SD from three independent experiments. GAPDH was used as a loading control. Full-length blots/gels are presented in Supplementary Figure 5.

To investigate the reasons for the disrupted cristae structures, we first focused on the MICOS components identified by IP (Fig. 1A,B). Knocking down any component of the complex, which contains Mitofilin, Sam50, and CHCHD 3 and 6, has been found to disrupt cristae structure^10^,^11^,^12^,^20^. Immunoblot results showed that knockdown of Mitofilin led to great reductions in Sam50 and CHCHD 3 and 6 (Fig. 4A), whereas CHCHD6 knockout did not affect Mitofilin, Sam50 or CHCHD 3 (Fig. 4B).

OPA1 has been reported to play vital roles not only in mitochondrial fusion^25^ but also in cristae remodeling^26^. Furthermore, OPA1 has been reported to form a complex with Mitofilin and CHCHD3 at CJs^11^. Immunoblot analysis revealed that OPA1 was greatly reduced in Mitofilin-deficient cells (Fig. 4A), whereas its expression was unaltered in CHCHD6-knockout cells (Fig. 4B). To exclude the possibility of a general decrease in mitochondrial protein levels in Mitofilin-knockdown cells, we assessed the protein levels of ATP5A1 (ATP synthase, H^+^ transporting, mitochondrial F1 complex, alpha subunit 1) in the IMM and Tom40 in the OMM. Western blot results revealed that the levels of these proteins were unchanged (Fig. 4A). Based on reports that Mitofilin and OPA1 play roles in the formation of CJs^26^,^27^, our results suggest that the cristae disruptions in Mitofilin-deficient cells may be attributed to the loss of proteins forming CJs. Thus, we conclude that the altered cristae structure in Mitofilin-knockdown cells is due to the disintegration of the MICOS complex, whereas CHCHD6 plays a distinct role in regulating cristae morphology.

### CHCHD6 interacts with OPA1 and physically binds to Sam50 via its N terminus

CHCHD 3 and 6 are identified as twin CX_9_C proteins with similar sizes and an overall sequence identity of 36%^8^. CHCHD3 has been characterized as a scaffolding protein for Sam50 in the OMM and the Mitofilin and OPA1 complex in the IMM^11^. CHCHD3 is supposed to bind to Mitofilin through the CHCH domain and to Sam50 through its N terminus. We investigated whether CHCHD6 plays similar roles as CHCHD3.

**Figure 5:**
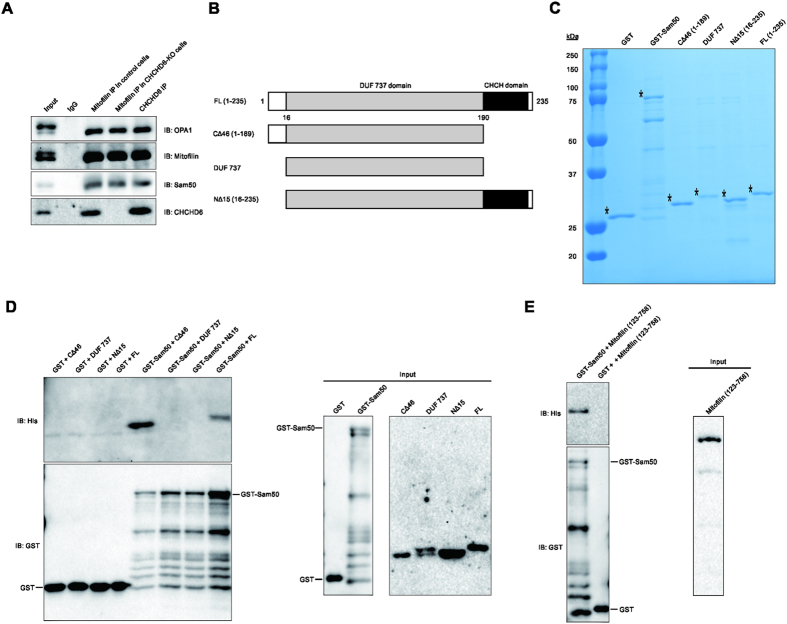
CHCHD6 interacts with OPA1 and directly interacts with Sam50. (**A**) The IP samples of Mitofilin and CHCHD6 were analyzed via SDS-PAGE followed by immunoblotting (IB) with the indicated antibodies. (**B**) Schematic illustration of the full-length and deletion variants of the CHCHD6 protein. (**C**) Coomassie Blue staining of purified GST, GST-Sam50, and His-tagged full-length and deletion variants of CHCHD6. The protein products are indicated by *asterisks*. (**D**) Mapping of the CHCHD6 and Sam50 interaction region on CHCHD6. *Left*, purified His-tagged full-length CHCHD6 and deletion variants were incubated with GST or GST-tagged Sam50. Then, GST pull-downs were conducted. *Right*, western blot analysis of 1% input of purified GST, GST-Sam50, and His-tagged full-length and deletion variants of CHCHD6. E, Sam50 directly interacts with Mitofilin. *Left*, the GST pull-down was performed by incubating His-tagged Mitofilin^123–758^ with GST-tagged Sam50 or GST. The pull-down protein products were analyzed by Western blot using anti-GST and anti-His antibodies. *Right*, western blot analysis of 1% input of His-Mitofilin^123–758^. Full-length blots/gels are presented in Supplementary Figure 6.

We performed IP experiments with CHCHD6 antibody in HeLa cells and immunoblot results showed that CHCHD6 interacts with soluble form but not long form of OPA1 (Fig. 5A). In accordance with a previous report^11^, we confirmed that Mitofilin only binds to soluble OPA1 (Fig. 5A).

It has been demonstrated that CHCHD6 physically interacts Mitofilin via its C terminus^12^. In the present study, we sought to examine whether CHCHD6 directly interacts with Sam50 *in vitro*. For this purpose, we performed glutathione S transferase (GST)-pull-down assays using GST-tagged Sam50. Expression vectors carrying GST, GST-tagged Sam50 and His-tagged CHCHD6 were constructed and independently introduced into *E. coli*. Coomassie Blue staining of the purified proteins (denoted by an *asterisk*) is shown in Fig. 5C. Our results revealed that purified Sam50 directly interacts with CHCHD6 (Fig. 5D).

Next, we set out to determine which region of CHCHD6 was responsible for Sam50 binding. CHCHD6 is predicted to have a myristoylation site and a DUF 737 (domain of unknown function) domain at the N-terminus and a CHCH domain at its C-terminus^12^ (Fig. 5B). We generated one variant of CHCHD6 containing the DUF 737 domain (DUF 737) and two deletion variants of CHCHD6 that lacked either the N-terminal 15 amino acids (NΔ15) or the C-terminal 46 amino acids (CΔ46). These variants were independently cloned into a bacterial expression vector with a His tag at their N-termini, expressed in *E. coli* and then purified. Coomassie Blue staining of the purified CHCHD6 variants (denoted by an *asterisk*) is shown in Fig. 5C. The GST pull-down assay was carried out using purified GST-tagged Sam50 protein and purified CHCHD6 variants. The results showed that the NΔ15 variant, but not the DUF 737 and CΔ46 variants of CHCHD6, interacted with Sam50 (Fig. 5D). Taken together, our results demonstrate that CHCHD6 physically interacts with Sam50 via its N terminus.

### Mitofilin directly binds to Sam50

Because knocking out CHCHD6 disorganized the cristae structure without affecting other cristae-sustaining proteins, we then evaluated whether CHCHD6 knockout affects the connection between Mitofilin and Sam50 in the OMM or soluble OPA1 in the IMS. IP experiments were performed using Mitofilin antibodies in control and CHCHD6-knockout cells, and immunoblot results revealed no change in CHCHD6-knockout cells and control cells (Fig. 5A). This result might due to a compensatory effect of CHCHD3 in connecting Sam50 and Mitofilin^11^ to Mitofilin binding Sam50 directly.

In yeast, Fcj1 (also known as Mitofilin in mammals) has been shown to directly bind the SAM/TOB complex component Tob55^17^, also known as Sam50 in mammals. Thus, in the present study, we sought to investigate whether Mitofilin physically interacts with Sam50 in mammals. To this end, we expressed human GST-tagged Sam50 with and His-tagged Mitofilin^123–758^ in *E. coli* to perform a GST-pull-down assay. Immunoblot results showed that Mitofilin directly interacts with Sam50 (Fig. 5E).

### Depletion of Mitofilin, but not CHCHD6 knockout, leads to apparent mitochondrial dysfunction

**Figure 6:**
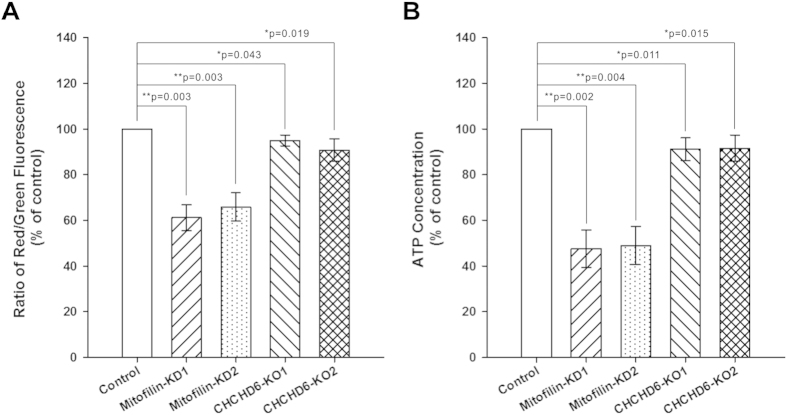
Effects of stable Mitofilin depletion and CHCHD6 knockout on mitochondrial function. (**A**) Effects of Mitofilin knockdown and CHCHD6 knockout on ΔΨm. ΔΨm was determined by the fluorescence ratio of red to green using the JC-1 assay. (**B**) Effects of Mitofilin knockdown and CHCHD6 knockout on intracellular ATP levels. Intracellular ATP production was measured using a luciferase-based assay; ATP levels were normalized to protein levels. Statistical analysis was performed using Student’s t-test (*P < 0.05 versus control, **P < 0.01 versus control). Each value represents mean ± SEM (n = 3).

Because knockdown of Mitofilin and knockout of CHCHD6 affected mitochondrial cristae morphology, we next determined whether Mitofilin knockdown and CHCHD6 knockout induce abnormal mitochondrial function. For this purpose, we evaluated mitochondrial membrane potential (ΔΨm) and levels of intracellular ATP content, which are indicators of mitochondrial activity. We performed the JC-1 assay to detect changes in ΔΨm. Our results (Fig. 6A) show that ΔΨm in Mitofilin-knockdown cells was clearly decreased compared with control cells whereas ΔΨm was only minimally changed in CHCHD6-knockout cells. We used a luciferase-based assay to estimate intracellular ATP content. As shown in Fig. 6B, intracellular ATP synthesis was greatly reduced in Mitofilin-knockdown cells but slightly decreased in CHCHD6-knockout cells. These results suggest that depletion of Mitofilin, but not knockout of CHCHD6, significantly affects mitochondrial function.

## Discussion

MICOS plays a dual role in maintaining cristae morphology and forming contact sites with the OMM to promote mitochondrial protein import into the OMM and IMS^7^. In our study, we immunoprecipitated two components of MICOS, Mitofilin and CHCHD6, in HeLa cells. IP and immunoblot results demonstrated that Mitofilin, Sam50, and CHCHD 3 and 6 are in the same complex. As Mitofilin and CHCHD6 are membrane-associated proteins, we took the following issues into consideration during IP experiments. An adequate number of cells need to be lysed to release enough protein to ensure effective IP. The selection of IP lysis buffer with suitable detergents at appropriate concentrations is also important, as the conformation of many membrane proteins can be affected by the type and concentration of detergents. Precautions such as preincubation of protein supernatant with protein A Sepharose beads before incubation with antibody-crosslinked beads and sufficient rinsing of the beads at each step can mitigate non-specific interactions.

Using two specific pairs of TALENs targeting the Mitofilin and CHCHD6 genes, we generated two cell clones in which Mitofilin was stably knocked down and two cell lines with CHCHD6 knockout. In Mitofilin-deficient cells, we observed vesicle-like cristae structures, and immunoblotting indicated that the altered cristae were due to great reductions in proteins that regulated cristae structure. CHCHD 3 and 6 lack mitochondrial targeting sequences and are supposed to be imported into mitochondria via the MIA pathway^28^. CHCHD3 requires the CHCH domain cysteines for assembly into macromolecular complexes following import into the IMS, and the CHCH domain of CHCHD3 is essential for binding to Mitofilin^29^. Mitofilin was found to directly interact with the C-terminus of CHCHD6, which includes the CHCH domain^12^. These reports indicate that Mitofilin is important for the assembly of CHCHD 3 and 6.

The TIM23 complex handles the import of precursor proteins containing N-terminal targeting signals into the IMM^13^. Here, we detected the protein levels of Tim23, the core component of the TIM23 complex, and found that it was slightly reduced in Mitofilin-deficient cells (Supplementary Fig. S2). IP experiments with Mitofilin antibody in HeLa cells revealed that Tim23 is a novel Mitofilin-binding protein (Supplementary Fig. S2), suggesting that Mitofilin might cooperate with Tim23 to promote mitochondrial protein import. In conclusion, loss of Mitofilin affected the assembly or stability of mitochondrial proteins, which could explain the decreased mitochondrial protein levels and thus the altered cristae morphology. At the same time, the reduced interaction of Mitofilin and Sam50 caused by their decreased protein levels in Mitofilin deficient cells may also lead to disrupted cristae structure.

Mitochondria in CHCHD6-knockout cells showed less cristae content than control cells. However, we could not find reduced protein levels of its binding partners. Knocking down Sam50 leads to the complete loss of cristae without affecting CHCHD3 and Mitofilin protein levels^20^, which suggests that Sam50 is an indispensable contact site of MICOS in the OMM for cristae maintenance. Our results showed that CHCHD6 directly interacts with Sam50 via its N-terminus. Given the unchanged protein levels of other MICOS components and the interaction between Mitofilin and Sam50 in CHCHD6 knockout cells, we speculate that the bridging of Sam50 in the OMM and MICOS in the IMM by CHCHD6 is indispensable for cristae maintenance.

**Figure 7:**
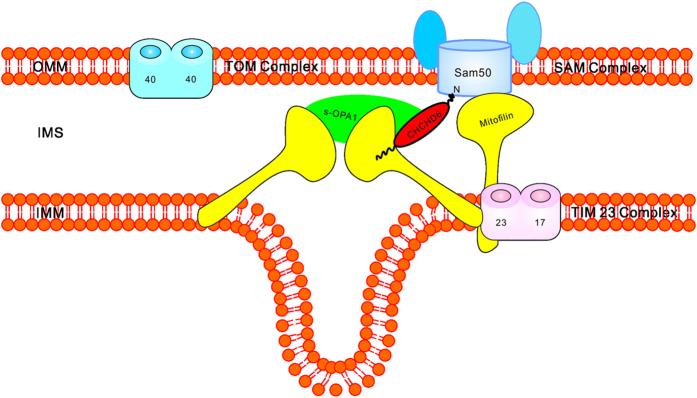
Proposed model of how Mitofilin and CHCHD6 function in cristae formation and preservation. Mitofilin and CHCHD6 forms a complex with OPA1 at CJs thereby influencing CJs formation and stability. Mitofilin and CHCHD6 directly connects MICOS with Sam50 would sustain cristae architecture. The direct interaction between Mitofilin and CHCHD6 was reported by Jie An, *et al.*^12^.

Although the mechanism by which cristae form remains to be explored, our results support the “cristae fission–fusion model”^3^ in which cristae propagation occurs as a result of cristae vesicles budding off from preexisting cristae and re-fusing with the inner membrane at a different site within the mitochondrion. We propose that the formation and maintenance of cristae requires contact sites in both the OMM and IMM and that scaffolding proteins bridge these sites (Fig. 7 *model*). First, proteins in the IMM, such as Mitofilin and OPA1, are responsible for the formation and stabilization of CJs. Second, proteins in the OMM, such as Sam50, offer contact sites for MICOS to sustain cristae structure. Third, scaffolding proteins, such as Mitofilin and CHCHD6, are responsible for bridging both contact sites in both the IMM and OMM.

We observed a clear decrease in ΔΨm and intracellular ATP content in Mitofilin-knockdown cells, which could be explained by the obvious defect in cristae morphology and drastic reductions of Mitofilin-binding proteins related to mitochondrial function. In contrast, ΔΨm and intracellular ATP production appeared to be minimally affected in CHCHD6-knockout cells. A recent study demonstrated that loss of CHCHD3 in HeLa cells slightly decreases total cellular ATP synthesis. As CHCHD3 has similar characteristics to CHCHD6^8^ and the CHCHD3 protein level was unchanged in CHCHD6-knockout cells, we speculate that CHCHD3 and CHCHD6 could compensate for each other to maintain mitochondrial function.

Taken together, we showed that both Mitofilin and CHCHD6 physically connect MICOS with Sam50 and Mitofilin is crucial for the assembly or stability of mitochondrial proteins involved in CJs formation and cristae maintenance. We propose that the integrity of MICOS and its efficient interaction with Sam50 are indispensable for cristae organization, which is relevant to mitochondrial function. In the future, it would be interesting to test whether or how Mitofilin promotes mitochondrial protein import by interacting with Sam50 or Tim23.

## Materials and Methods

### TALEN design and construction

Exons of human Mitofilin or CHCHD6 and their immediate flanking regions were scanned for putative TALEN binding pairs using TAL Effector Nucleotide Targeter 2.0^30^ with the following constraints: (1) spacer length of 14–20 bp, (2) repeat array length of 14–21 bp, and (3) an upstream base of T only. Based on these guidelines, a sample sequence was selected for a pair of TALENs to target the third exon of the human Mitofilin gene, with 5'-ACTCAGACAAACTCTTCG-3' as the spacer. The left TALEN sequence was 5'-AGAGAAAACCATACCTT-3’, and the right sequence was 5'-GCAGGACCAAGAACCATCT-3' A pair of TALENs was designed to target the second exon of human CHCHD6 gene, with 5'-ACCTTTGGCCTTCA-3' as the spacer. The left TALEN sequence was 5'-TGCTCCCACATCTTCT-3', and the right sequence was 5'-TCTCAAGTTGCCATCT-3'. All coding sequences of the binding pairs Mitofilin/CHCHD6-L and Mitofilin/CHCHD6-R were cloned into the pUC18 vector and further moved to pCS2-PEAS and pCS2-PERR using *Spe* I and *Nhe* I, yielding pTALEN-Mitofilin/CHCHD6-L and pTALEN-Mitofilin/CHCHD6-R.

### Cell culture and screening

HeLa cells were maintained in Dulbecco’s modified Eagle’s medium (DMEM) supplemented with 10% (vol/vol) fetal bovine serum (Hyclone) at 37 °C in a 5% (vol/vol) CO_2_ atmosphere. Cells were transfected with a mixture of pTALEN-L and pTALEN-R at a ratio of 1:1. Cells were harvested 2–3 d after transfection to extract genomic DNA for PCR-amplification of the targeted exon. If the targeted gene was disrupted, as confirmed by Sanger sequencing, cells were trypsinized, resuspended in culture medium, and then allocated to 96-well plates, with each well harboring one cell. After two weeks, cell clones were expanded, and whole-cell extracts were analyzed by SDS-PAGE and immunoblotting. For transfection experiments, Lipofectamine 2000 and Opti-MEM I (Invitrogen, Carlsbad, CA, USA) were used for transient transfection with expression constructs according to the manufacturer’s protocol.

### Plasmids and antibodies

Plasmids containing the coding sequences of mono-/di- and tri-TALE repeats used in Unit Assembly and pCS2-PEAS and pCS2-PERR, SP6-driven expression vectors containing the coding sequences of Fok I and the N-/C-termini of TALEN, were purchased from Cowin Biotech Co., Ltd., China. Full-length human Sam50 cDNAs were cloned into the pGEX-6P-1 (GE Healthcare) vector. Human Mitofilin^123–758^ and full-length human CHCHD6 and its variants were cloned to the pET-28a (Novagen) vector. Primers used to construct GST-tagged and His-tagged proteins are shown in Supplementary Table S2. The following primary antibodies were used: rabbit polyclonal anti-Mitofilin (10179-1-AP, Proteintech); rabbit polyclonal anti-CHCHD6 (20639-1-AP, Proteintech); rabbit polyclonal anti-Tim23 (11123-1-AP, Proteintech); rabbit monoclonal anti-Sam50 (ab133709, Abcam); rabbit polyclonal anti-Tom40 (18409-1-AP, Proteintech); rabbit polyclonal anti-CHCHD3 (25625-1-AP, Proteintech); rabbit polyclonal anti-GST (10000-0-AP, Proteintech); mouse monoclonal anti-His (66005-1-Ig, Proteintech); rabbit polyclonal anti-ATP5A1 (14676-1-AP, Proteintech); rabbit monoclonal anti-OPA1 (7529-1, Epitomics); and rabbit polyclonal anti-GAPDH (10494-1-AP, Proteintech).

### Electron microscopy

Cells were fixed for 1 h at 4 °C in phosphate buffer (PB) containing 2.5% glutaraldehyde. After five washes in PB buffer (pH 7.4) for 5 min each, cells were post-fixed for 40 min at RT in 1% osmium tetroxide (OSO_4_). After further washes with PB, cells were dehydrated in 70%, 80%, 95% and 100% ethanol (10 min per step), and the cells were then further dehydrated in 100% acetone twice for 10 min each at RT. The samples were then infiltrated in 1:1 (vol/vol) acetone/epoxy resin (30 min), 1:3 acetone/epoxy resin (30 min), 100% epoxy resin (3 h) and finally 100% epoxy resin (48 h) at 60 °C for polymerization. The sections were supported on copper grids. The 80-nm sections were post-stained in Sato lead for 1 min, and the stained sections were imaged onto negatives using a transmission electron microscope (FEI Tecnai G2 20 TWIN) operated at 160 kV. Scanning transmission electron microscopy (STEM) tomography was performed on a 300-kV field-emission transmission electron microscope (FEI Titan Krios) in STEM mode with a Fischione Model 3000 annular dark field detector for dark-field imaging. No colloidal gold particles were used as an alignment marker. The single-axis tilt series of 800-nm sections was collected from −64° to +64° with a step size of 2°, a semi-conversion angle of 4.3 mrad, a probe current of 7.4 pA, a dwell time of 8 μsec, a camera length of 173 mm, and a pixel size of 1.75 nm.

### IP assay

IPs were performed as previously described^31^. Briefly, 4 μg of Mitofilin, CHCHD6 or negative control antibody was crosslinked to protein A Sepharose beads using dimethylpimelimidate (Sigma) at a 1:1 ratio of beads to antibody. HeLa cells were lysed in IP lysis buffer (Beyotime, China) containing protease inhibitor EDTA-free (Roche) for 30 min at 4 °C followed by centrifugation at 14 000 g for 30 min at 4 °C. The supernatant was then incubated overnight with 50 μl antibody-crosslinked beads at 4 °C. The immunocomplex was collected, washed five or six times with cold PBS, and mixed with protein gel loading buffer to elute the proteins at room temperature. Mitofilin and CHCHD6 immunoprecipitates were trichloroacetic acid (TCA) precipitated and sent to BGI TechSolutions Co., Ltd., Shenzhen, China, for LC-MS/MS analysis.

### GST pull-down assay

GST-Sam50 and GST-Mitofilin were expressed in *E. coli* (BL21) and purified by binding to glutathione agarose (Millipore). His-CHCHD6 and His-tagged variants of CHCHD6 were expressed in *E. coli* (BL21) and purified by binding to Nickel-NTA agarose (Novagen). A total of 10 μg purified GST-tagged protein, 10 μg His-tagged protein and 40 μl glutathione Sepharose 4B were mixed in 1 ml PBS containing protease inhibitor EDTA-free (Roche). The mixture was rotated overnight at 4 °C and washed five times with cold PBS. To elute the proteins, 80 μl of protein gel loading buffer was added to the beads, which were then boiled for 10 min.

### Detection of mitochondrial membrane potential

A mitochondrial membrane potential assay kit with JC-1 (Beyotime, China) was employed to detect the ΔΨm according to the manufacturer’s instructions. Briefly, after the indicated treatments, cells were loaded with JC-1 for 30 min at 37 °C. Cells were then rinsed twice with medium and resuspended in fresh medium. Fluorescence was monitored with a fluorescence microscope (Infinite200 PRO, TECAN, Switzerland). The ΔΨm was calculated as the fluorescence ratio of red to green.

### Measurement of intracellular ATP levels

Intracellular ATP levels were measured using an ATP assay kit (Beyotime, China) according to the manufacturer’s directions. After the indicated treatments, cells were lysed and centrifuged at 12,000 g for 5 min at 4 °C. Supernatants (20 μL) were mixed with 100 μL of ATP detection working solution in a white 96-well plate. Luminescence (RLU) was measured using a fluorescence microscope (Infinite200 PRO, TECAN, Switzerland). Standard curves were generated, and protein concentration was determined using a Bradford protein assay. ATP levels were normalized to protein levels.

### Statistical analysis

Statistical analysis was performed using one-tailed Student’s t test. Data are expressed as mean ± standard error of the mean (SEM). A value of P < 0.05 was considered as statistically significant.

## Additional Information

**How to cite this article**: Ding, C. *et al.* Mitofilin and CHCHD6 physically interact with Sam50 to sustain cristae structure. *Sci. Rep.* **5**, 16064; doi: 10.1038/srep16064 (2015).

## Supplementary Material

Supplementary Information

Supplementary Table S1

Supplementary Table S2

## References

[b1] Perkins G. *et al.* Electron tomography of neuronal mitochondria: three-dimensional structure and organization of cristae and membrane contacts. J Struct Biol 119, 260–272 (1997). 9245766 10.1006/jsbi.1997.3885

[b2] Reichert A. S. & Neupert W. Mitochondriomics or what makes us breathe. Trends Genet 20, 555–562 (2004). 15475115 10.1016/j.tig.2004.08.012

[b3] Zick M., Rabl R. & Reichert A. S. Cristae formation—linking ultrastructure and function of mitochondria. Bba-mol Cell Res 1793, 5–19 (2009). 10.1016/j.bbamcr.2008.06.013 18620004

[b4] Trimmer P. A. *et al.* Abnormal mitochondrial morphology in sporadic Parkinson's and Alzheimer's disease cybrid cell lines. Exp Neurol 162, 37–50 (2000). 10716887 10.1006/exnr.2000.7333

[b5] Acehan D., Xu Y., Stokes D. L. & Schlame M. Comparison of lymphoblast mitochondria from normal subjects and patients with Barth syndrome using electron microscopic tomography. Lab Invest 87, 40–48 (2007). 17043667 10.1038/labinvest.3700480 PMC2215767

[b6] Arismendi-Morillo G. Electron microscopy morphology of the mitochondrial network in gliomas and their vascular microenvironment. BBA-Bioenergetics 1807, 602–608 (2011). 21692239 10.1016/j.bbabio.2010.11.001

[b7] Horvath S. E. *et al.* Role of membrane contact sites in protein import into mitochondria. Protein Sci 24, 277–297 (2015). 25514890 10.1002/pro.2625 PMC4353355

[b8] Zerbes R. M. *et al.* Mitofilin complexes: conserved organizers of mitochondrial membrane architecture. Biol Chem 393, 1247–1261 (2012). 23109542 10.1515/hsz-2012-0239

[b9] von der Malsburg K. *et al.* Dual role of mitofilin in mitochondrial membrane organization and protein biogenesis. Dev cell 21, 694–707 (2011). 21944719 10.1016/j.devcel.2011.08.026

[b10] John G. B. *et al.* The mitochondrial inner membrane protein mitofilin controls cristae morphology. Mol Biol Cell 16, 1543–1554 (2005). 15647377 10.1091/mbc.E04-08-0697 PMC551514

[b11] Darshi M. *et al.* ChChd3, an inner mitochondrial membrane protein, is essential for maintaining crista integrity and mitochondrial function. J Biol Chem 286, 2918–2932 (2011). 21081504 10.1074/jbc.M110.171975 PMC3024787

[b12] An J. *et al.* CHCM1/CHCHD6, novel mitochondrial protein linked to regulation of mitofilin and mitochondrial cristae morphology. J Biol Chem 287, 7411–7426 (2012). 22228767 10.1074/jbc.M111.277103 PMC3293568

[b13] Schmidt O., Pfanner N. & Meisinger C. Mitochondrial protein import: from proteomics to functional mechanisms. Nat Rev Mol Cell Bio 11, 655–667 (2010). 20729931 10.1038/nrm2959

[b14] Armstrong L. C., Komiya T., Bergman B. E., Mihara K. & Bornstein P. Metaxin is a component of a preprotein import complex in the outer membrane of the mammalian mitochondrion. J Biol Chem 272, 6510–6518 (1997). 9045676 10.1074/jbc.272.10.6510

[b15] Armstrong L. C., Saenz A. J. & Bornstein P. Metaxin 1 interacts with metaxin 2, a novel related protein associated with the mammalian mitochondrial outer membrane. J Cell Biol 74, 11–22 (1999). 10381257

[b16] Kozjak‐Pavlovic V. *et al.* Conserved roles of Sam50 and metaxins in VDAC biogenesis. EMBO Rep 8, 576–582 (2007). 17510655 10.1038/sj.embor.7400982 PMC2002532

[b17] Körner C. *et al.* The C-terminal domain of Fcj1 is required for formation of crista junctions and interacts with the TOB/SAM complex in mitochondria. Mol Biol Cell 23, 2143–2155 (2012). 22496419 10.1091/mbc.E11-10-0831 PMC3364178

[b18] Bohnert M. *et al.* Role of mitochondrial inner membrane organizing system in protein biogenesis of the mitochondrial outer membrane. Mol Biol Cell 23, 3948–3956 (2012). 22918945 10.1091/mbc.E12-04-0295 PMC3469511

[b19] Xie J., Marusich M. F., Souda P., Whitelegge J. & Capaldi R. A. The mitochondrial inner membrane protein mitofilin exists as a complex with SAM50, metaxins 1 and 2, coiled-coil-helix coiled-coil-helix domain-containing protein 3 and 6 and DnaJC11. FEBS Lett 581, 3545–3549 (2007). 17624330 10.1016/j.febslet.2007.06.052

[b20] Ott C. *et al.* Sam50 functions in mitochondrial intermembrane space bridging and biogenesis of respiratory complexes. Mol Cell Biol 32, 1173–1188 (2012). 22252321 10.1128/MCB.06388-11 PMC3295012

[b21] Bogdanove A. J. & Voytas D. F. TAL effectors: customizable proteins for DNA targeting. Science 333, 1843–1846 (2011). 21960622 10.1126/science.1204094

[b22] Mahfouz M. M. *et al.* *De novo*-engineered transcription activator-like effector (TALE) hybrid nuclease with novel DNA binding specificity creates double-strand breaks. Proc Natl Acad Sci USA 108, 2623–2628 (2011). 21262818 10.1073/pnas.1019533108 PMC3038751

[b23] Christian M. *et al.* Targeting DNA double-strand breaks with TAL effector nucleases. Genetics 186, 757–761 (2010). 20660643 10.1534/genetics.110.120717 PMC2942870

[b24] Yang R..-f. *et al.* Mitofilin regulates cytochrome c release during apoptosis by controlling mitochondrial cristae remodeling. Biochem Bioph Res Co 428, 93–98 (2012). 10.1016/j.bbrc.2012.10.012 23058921

[b25] Olichon A. *et al.* The human dynamin-related protein OPA1 is anchored to the mitochondrial inner membrane facing the inter-membrane space. FEBS Lett 523, 171–176 (2002). 12123827 10.1016/s0014-5793(02)02985-x

[b26] Frezza C. *et al.* OPA1 controls apoptotic cristae remodeling independently from mitochondrial fusion. Cell 126, 177–189 (2006). 16839885 10.1016/j.cell.2006.06.025

[b27] Mun J. Y. *et al.* Caenorhabditis elegans mitofilin homologs control the morphology of mitochondrial cristae and influence reproduction and physiology. J Cell Physiol 224, 748–756 (2010). 20578245 10.1002/jcp.22177

[b28] Banci L., Bertini I., Ciofi-Baffoni S. & Tokatlidis K. The coiled coil-helix-coiled coil-helix proteins may be redox proteins. FEBS Lett 583, 1699–1702 (2009). 19345215 10.1016/j.febslet.2009.03.061

[b29] Darshi M., Trinh K. N., Murphy A. N. & Taylor S. S. Targeting and import mechanism of coiled-coil helix coiled-coil helix domain-containing protein 3 (ChChd3) into the mitochondrial intermembrane space. J Biol Chem 287, 39480–39491 (2012). 23019327 10.1074/jbc.M112.387696 PMC3501047

[b30] Doyle E. L. *et al.* TAL Effector-Nucleotide Targeter (TALE-NT) 2.0: tools for TAL effector design and target prediction. Nucleic Acids Res 40, W117–W122 (2012). 22693217 10.1093/nar/gks608 PMC3394250

[b31] Das R. *et al.* SR proteins function in coupling RNAP II transcription to pre-mRNA splicing. Mol Cell 26, 867–881 (2007). 17588520 10.1016/j.molcel.2007.05.036

