# Supplementary material for: Mitofilin and CHCHD6 physically interact with Sam50 to sustain cristae structure
Source: Sci Rep. 2015 Nov 4;5:16064. doi: 10.1038/srep16064 (PMC4632003; doi:10.1038/srep16064)
Supplement: Supplementary Information [file srep16064-s1.doc]

**Supplementary Information for “****Mitofilin and CHCHD6 physically interact with Sam50 to sustain cristae structure”**

Chengli Ding ^1#^, Zhifei Wu ^1#^, Lei Huang ^1^, Yajie Wang ^1,2^, Jie Xue^1^, Si Chen^1^, Zixin Deng ^1^, Lianrong Wang ^1^, Zhiyin Song ^3^, Shi Chen ^1*^

^1^ Key Laboratory of Combinatorial Biosynthesis and Drug Discovery, Ministry of Education, and School of Pharmaceutical Sciences, Wuhan University, Wuhan, China

^2^ Taihe Hospital, Hubei University of Medicine, Shiyan, Hubei, China

^3^ College of Life Sciences, Wuhan University, Wuhan, China

# These authors contributed equally to this work.


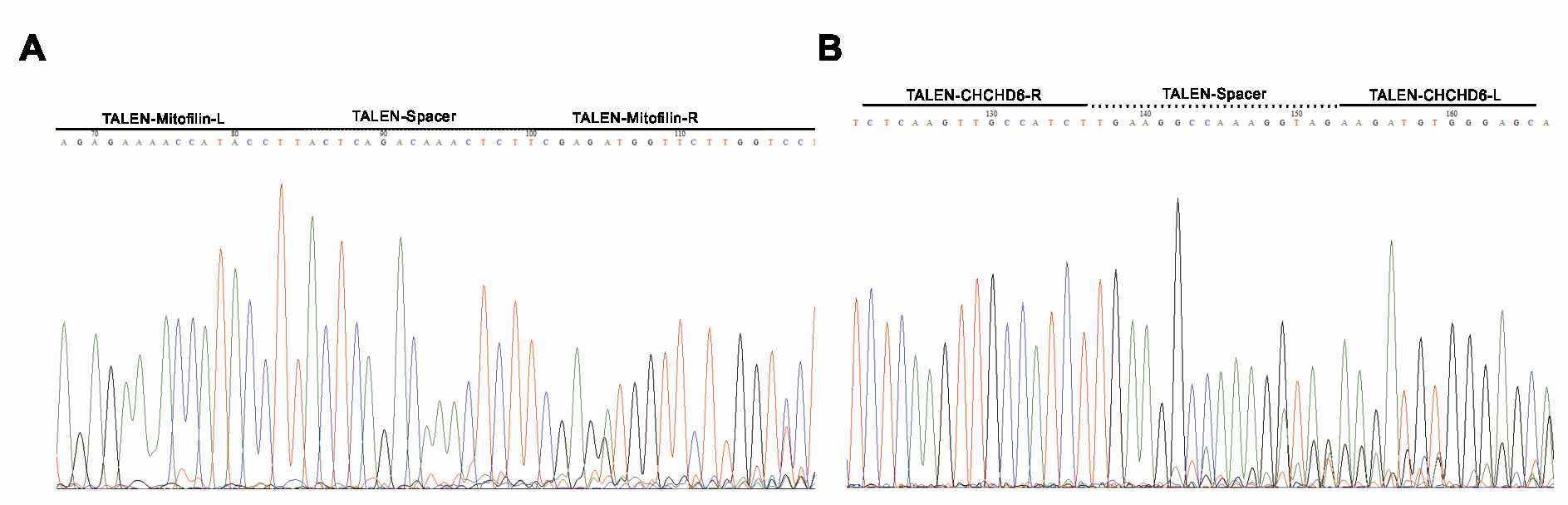


Fig. S1. Sanger sequencing of PCR-amplification of the targeted exon of Mitofilin (A) or CHCHD6 (B) confirmed that the designed TALEN binding pairs were efficient.


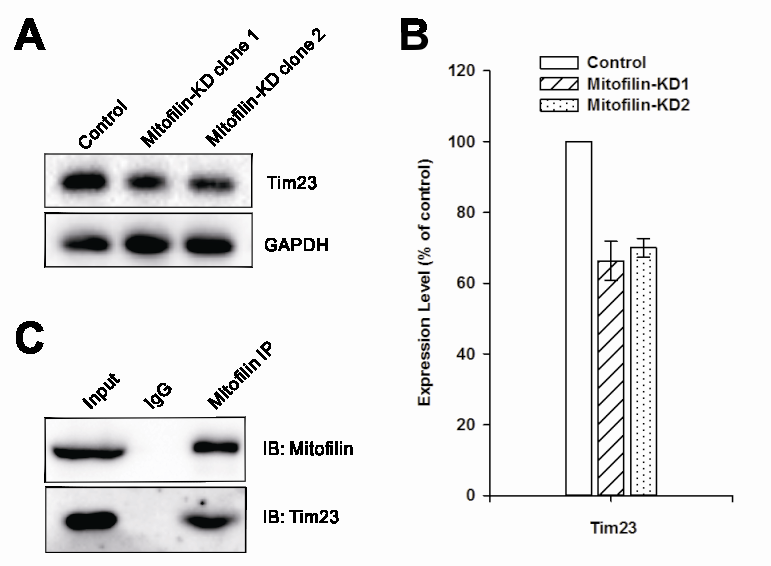


Fig. S2. Mitofilin interacts with Tim23, and Mitofilin-KD decreases the protein level of Tim23. (A and B) Equal amounts of protein samples in control cells and Mitofilin-knockdown cells were analyzed via SDS-PAGE followed by immunoblot with Tim23 antibody. The values represent the average protein expression ± SD from three independent experiments. GAPDH was used as a loading control. (C) The IP sample of Mitofilin was analyzed via SDS-PAGE followed by immunoblotting (IB) with Tim23 antibody. Full-length blots/gels are presented in Supplementary Figure 7.


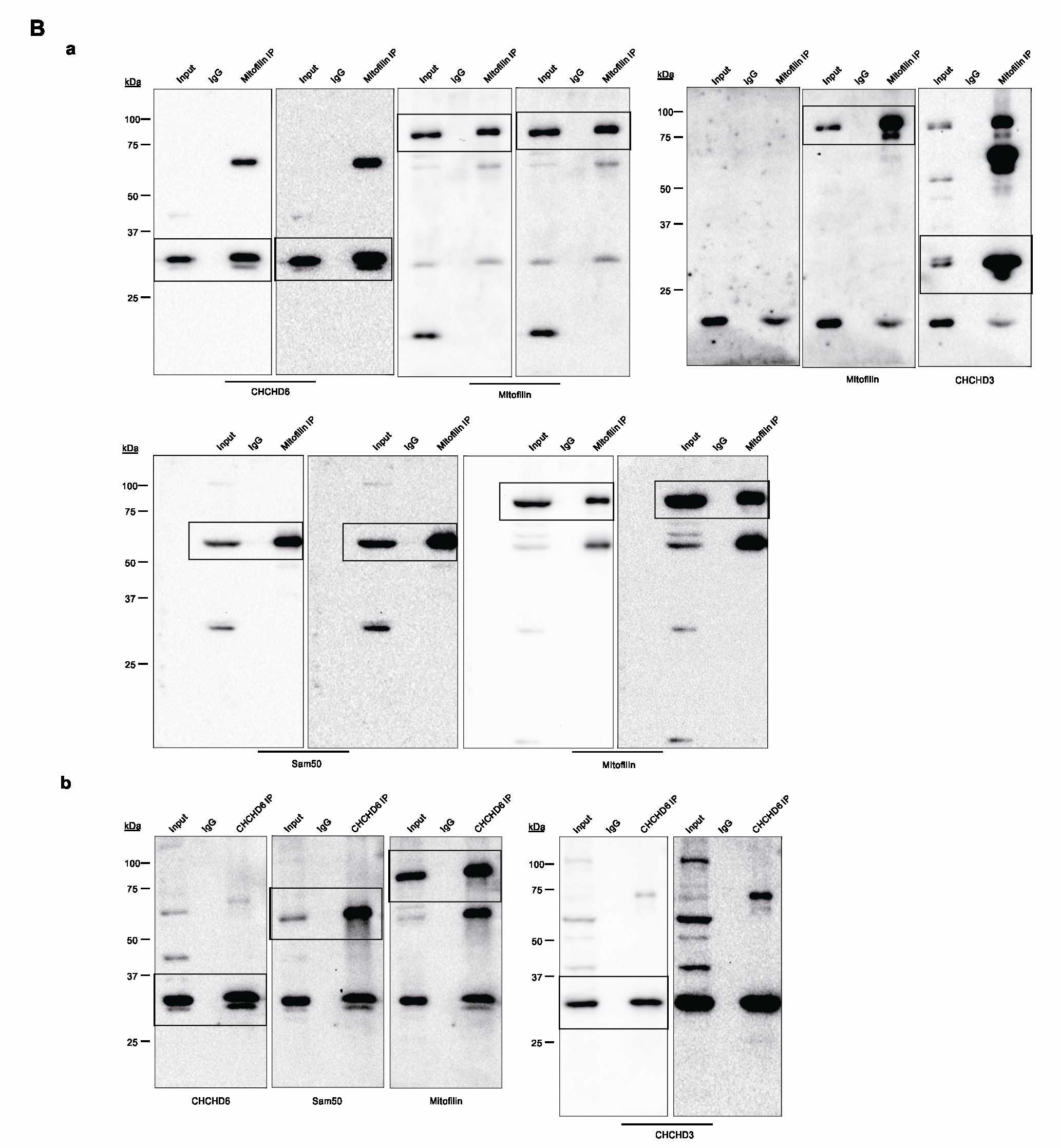


Fig. S3. Full-length blots/gels related to Figure 1.


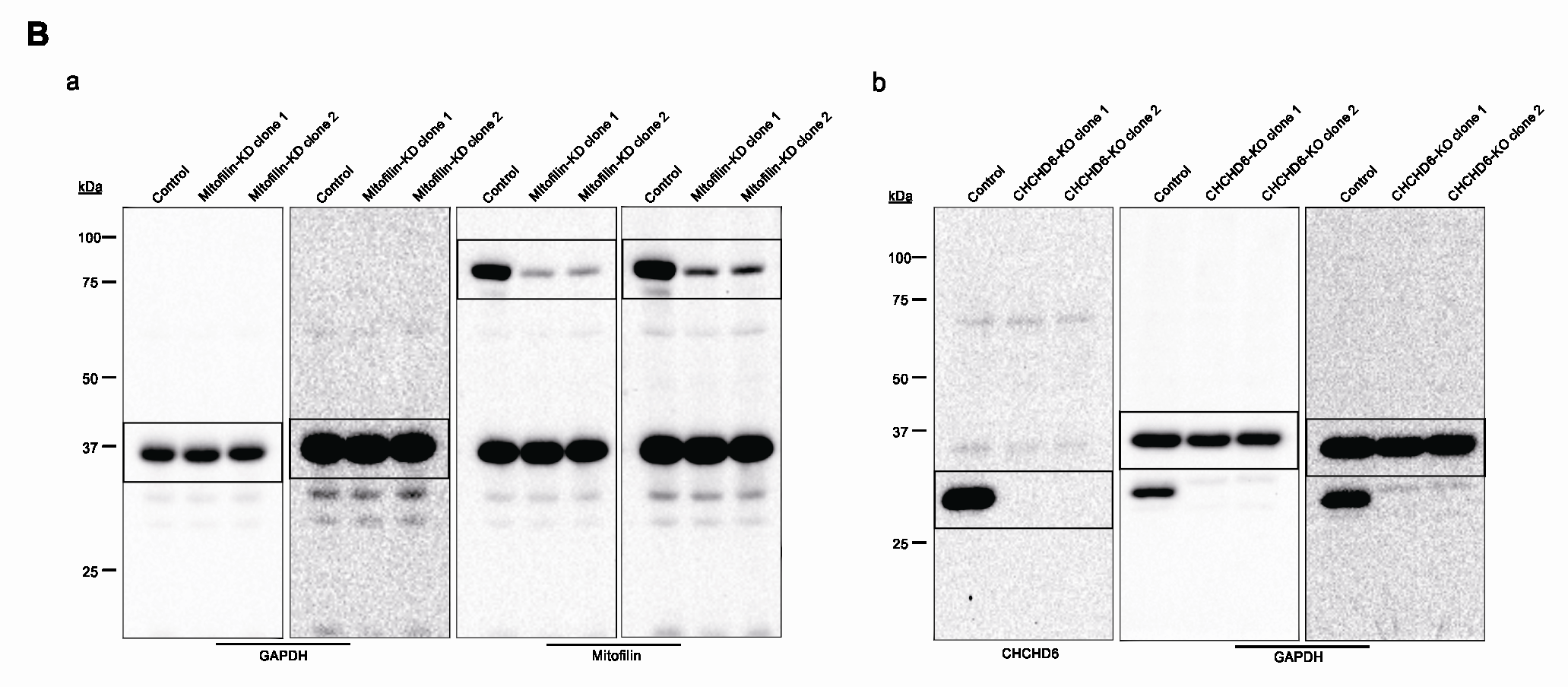


Fig. S4. Full-length blots/gels related to Figure 2


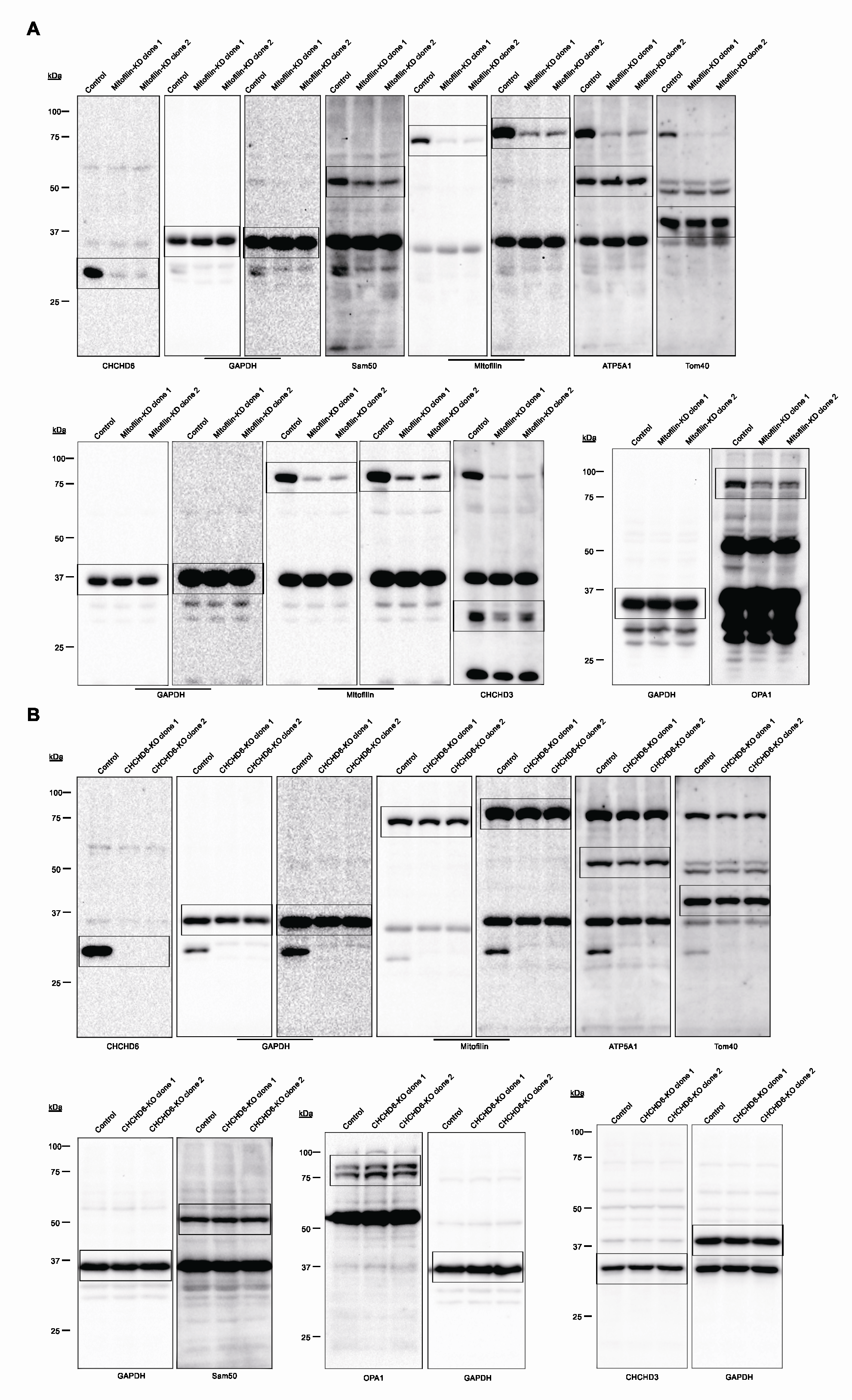


Fig. S5. Full-length blots/gels related to Figure 4.


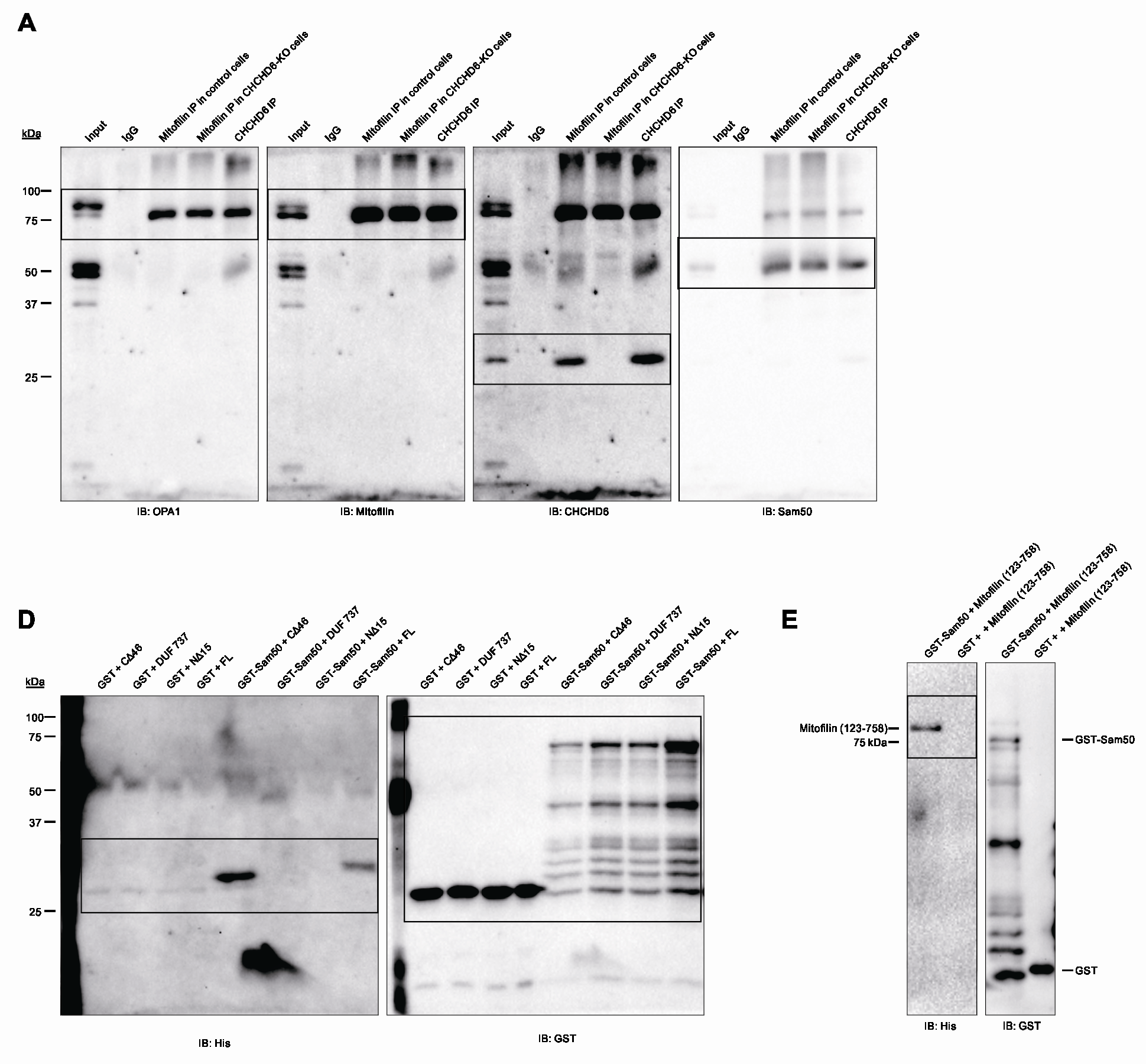


Fig. S6. Full-length blots/gels related to Figure 5.


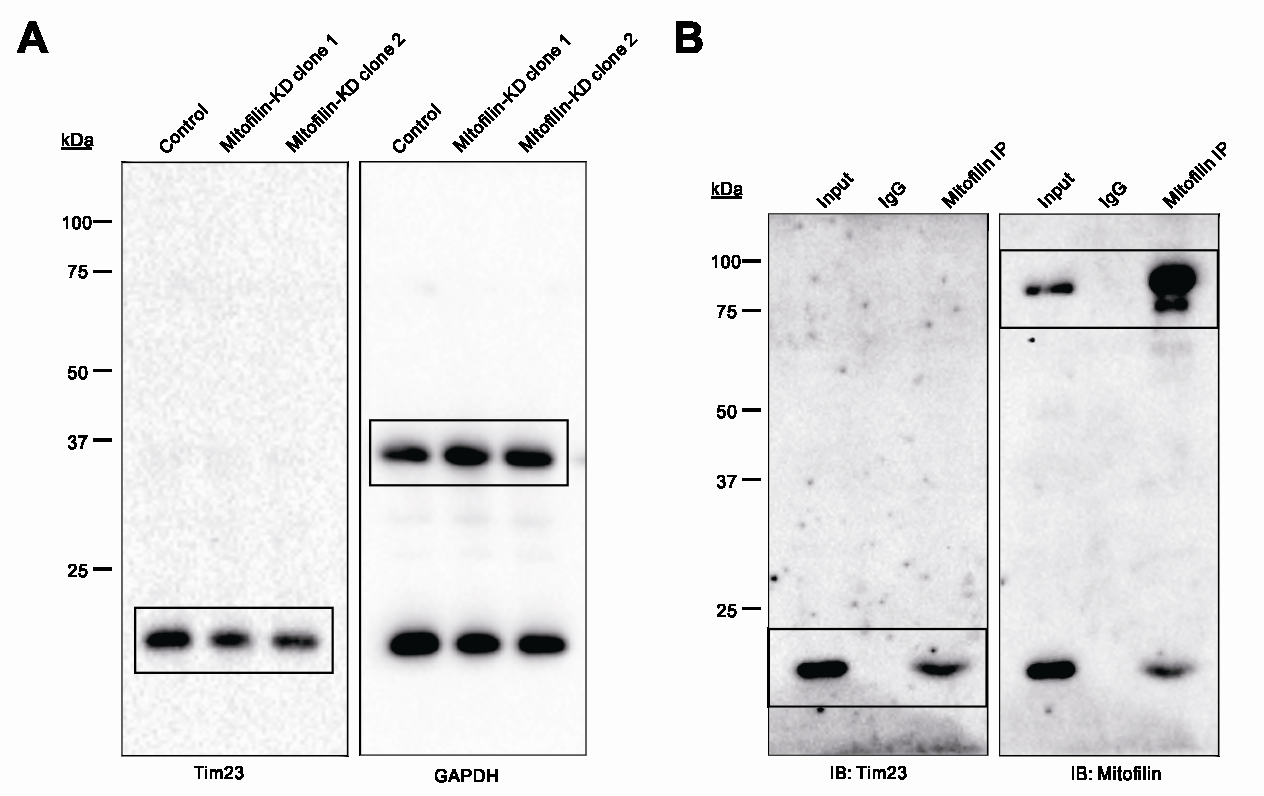


Fig. S7. Full-length blots/gels related to Supplementary Fig. S2.
